# Supplementary material for: Unpredictable benefits of social information can lead to the evolution of individual differences in social learning
Source: Nat Commun. 2024 Jun 15;15:5138. doi: 10.1038/s41467-024-49530-4 (PMC11180142; doi:10.1038/s41467-024-49530-4)
Supplement: Supplementary file 1 — Supplementary Information [file 41467_2024_49530_MOESM1_ESM.pdf]

Supplementary Information for

“Unpredictable benefits of social information can lead to the evolution of individual differences in social learning”

Pieter van den Berg, TuongVan Vu, Lucas Molleman

Contents:

- Supplementary Methods
- Supplementary Note 1
- Supplementary Figures
- Supplementary References

Supplementary Methods

Alternative implementation of developmental flexibility

We developed an alternative implementation of our model where the reliance on social learning does not change directly (as in our original model), but rather changes as a result of changing beliefs on the expected payoffs of individual and social learning, respectively.

At birth, individuals now have genetically determined initial expectations of the payoff of individual learning and of social learning, respectively denoted $E_{ind,0}$ and $E_{soc,0}$ (these are initialized randomly between -1 and 1 for each individual at the start of the simulation). The probability that an individual relies on social learning (S) now depends on these two expected payoffs through a softmax function, as follows:

$$S = \frac{1}{1 + e^{-b * (E_{ind} - E_{soc})}}$$

Where b, the 'temperature' of the softmax function, was a global parameter set to 4. After the individual has adopted a trait through either type of learning, they update the expected payoff of that type of learning as follows:

$$E_{new} = E_{old} + \Delta * (\pi - E_{old})$$

Where Δ is the individual's flexibility and π is the actual payoff of the adopted trait. As in the original model, flexibility (Δ) is a genotypically determined evolving variable. Here it can be thought of as a 'learning rate' as used in standard models of reinforcement learning[1].

We used this alternative model to run simulations for the scenario 1 from the main text (impact of effectiveness on social learning, see Fig 2). The results closely matched the results of the original model, and are depicted in Fig S2.

Supplementary Note 1

Evaluation of the effectiveness of both types of learning across the scenarios of our model

In the evolutionary simulations presented in the main text, natural selection can respond to individual variation in the effectiveness of social learning. That is, developmental flexibility may be favoured if there are some individuals for whom social learning tends to lead to better outcomes than individual learning, while for others, individual learning tends to lead to better outcomes than social learning.

In this Supplementary Note, we will characterise the effectiveness of individual and social learning based on the probabilities that individual and social learning will lead to 'correct' decisions: to adopt beneficial traits and to avoid adopting detrimental traits. The payoffs that are associated with cultural variants in our model are always equal to 1 or -1. Since our model is entire symmetrical with respect to positive and negative payoffs, we will for simplicity define effectiveness (denoted *P*) as the probability that an individual who is considering a cultural trait that has a positive payoff in fact adopts this trait. Below, we will calculate *P* for both individual learning (P_{ind}) and for social learning (P_{soc}) across the scenarios we considered in the main text.

Across our scenarios, P_{ind} is constant. This means that we only need to describe P_{soc} for each scenario. In this section, we will show that the parameter ranges favouring the evolution of developmental flexibility in reliance on social learning in our simulations are indeed those where for some individuals $P_{ind} > P_{soc}$, while for other individuals $P_{ind} < P_{soc}$. The code for producing the graphs below can be found in the repository associated with this paper (https://osf.io/7ta9m/).

Individual learning

In our model, individual learning is operationalised by randomly drawing an assessed payoff from a normal distribution with a mean that is equal to the actual payoff, and a standard deviation of 1. For a beneficial trait (with a payoff +1), the probability that a randomly chosen value from this distribution yields a positive number is given by the cumulative distribution function (cdf) of the normal distribution: $P_{ind} = 0.5 * \left[1 + \mathrm{erf}\left(\frac{1}{\sqrt{2}}\right)\right] = 0.84$, where *erf* is the Gauss error function. Hence, across all scenarios that we investigated, individual learning always has a probability of 0.159 to lead to a failure to adopt a beneficial trait (or the adoption of a detrimental trait).

Social learning

The description of P_{soc} varies between scenarios, but the logic will be similar across them. For any cultural trait, individuals take a sample of *M* others and assess the payoffs that the others obtain for expressing it. Below we will discuss each of the scenarios in turn.

Scenario 1

In scenario 1, we varied the effectiveness of social learning by varying the standard deviation of the noise on payoff assessment (denoted sd_{soc}). We varied both the average social learning effectiveness and the degree of individual variation in social learning effectiveness. Let σ_{soc} indicate the assessment error in social learning, which is equal to $\frac{sd_{soc}}{\sqrt{M}}$.

Similar to the expression of P_{ind}, we can calculate the probability that social learning of a beneficial trait indeed yields an assessment that the payoff is positive using the cumulative distribution function (cdf) of the normal distribution with mean=1 and s.d. = σ_{soc}:

$$P_{soc} = 0.5 * \left[1 + \text{erf}\left(\frac{1}{\sigma_{soc} * \sqrt{2}}.\right)\right] = 0.84$$

From the expressions above, we can see that $P_{ind} < P_{soc}$ if $sd_{soc} < \sqrt{M}$. This makes clear that the scope for social learning to outperform individual learning decreases with error in social learning (sd_{soc}) and increases with the root of the number of sampled others (M). Figure SN1 illustrates the relative performance of social and individual learning as a function of sd_{soc}.

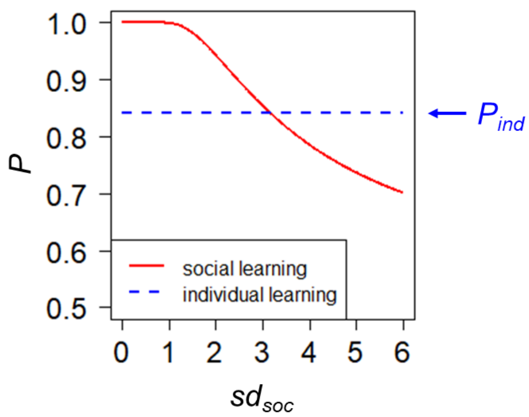

Fig. SN1. Performance of social learning (P_{soc}) as a function of individuals' social assessment accuracy (sd_{soc}), for M=10 (red line). The dashed blue line indicates the performance of individual learning (P_{ind}).

Fig. SN1 can help understand the results in Fig. 2a,b of the main text. In populations where average effectiveness of social learning is high (Fig. 2a,b; purple lines), sd_{soc} is small (on average 0.1) and therefore $P_{soc} > P_{ind}$ for basically all individuals. In populations where average effectiveness of social learning is low (Fig. 2a,b; blue lines), sd_{soc} is large (on average 5.0) and $P_{soc} < P_{ind}$, for all individuals. Crucially, if average effectiveness of social learning is intermediate (Fig. 2a,b; yellow lines; average sd_{soc} is 3.5), social learning and individual learning are on average about equally effective. If there is individual variation in sd_{soc} (further to the right in Fig. 2a,b) around this average, this means that some individuals in a population have a value of sd_{soc} to the left of the intersection point ($P_{soc} > P_{ind}$), and other individuals have a value to the right of the intersection point ($P_{soc} < P_{ind}$). As a result, developmental flexibility in reliance on social learning may be favoured by natural selection.

Scenario 2

In scenario 2, individuals receive different payoffs from adopting cultural traits. This means that payoffs of self and others can be 'mismatched': individuals may observe others express a trait and get positive payoffs (+1), and themselves receive a negative payoff from it (-1), and *vice versa*. We operationalize variation in payoffs by drawing, for each individual, a random number from a beta distribution. This number determines which five out of ten traits yield a positive payoff (giving rise to six 'types' that have positive payoffs for k=0, 1, 2, 3, 4 or 5 out of the five odd (as opposed to even) traits; see Methods in the main text for details). The parameters of this beta distribution (α and β) determine how much the payoffs for cultural traits vary in the population.

To calculate P_{soc} in this scenario, we first define a 'proximity matrix' K that indicates the proportion of beneficial and detrimental traits each pair of types have in common. Each cell in this matrix can be calculated as $K_{ij} = \frac{k_i * k_j + (n-k_i)*(n-k_j)}{n^2}$, where n is the number of odd and even traits (note that in our simulations, n=5, for a total of 10 traits). This expression can be derived as follows. For each odd trait, the probability that two individuals of types k_i and k_j have a positive payoff is given by k_i/n (the probability that the individual of type *i* receives a positive payoff for it) times k_j/n (the probability that the individual of type *j* receives a positive payoff for it). Similarly, for each even trait, this probability is $(n-k_i)/n$ times $(n-k_j)/n$. For the expected number of common traits for individuals of types *i* and *j*, we simply take the sum across odd and even traits, to arrive at K_{ij}. (Note that under our assumption of an equal number of beneficial and detrimental traits, the expressions for detrimental traits are complementary to the expressions above. The resulting factor '2' in both the numerator and denominator cancels out in the expression for K_{ij}.)

For each type k_i, the expected proportion of an individual observing 'matched' (as opposed to 'mismatched') traits in social learning is $p_i = \sum_{j=0}^{n} f_j * K_{ij}$, where f_j denotes the frequency of each type *j* in the population. This frequency is given by $f_j = I(\alpha, \beta)_{j+1} - I(\alpha, \beta)_j$ where $I(\alpha, \beta)$ is the cdf of the beta function (the regularised incomplete beta function).

When individuals learn socially, they may observe a mix of 'matched' and 'mismatched' cases. In mismatched cases, the social information is likely to point in the wrong direction, but there is still a small probability that it points in the right direction. However, on the whole, social information points in the right direction more often in matched cases than in mismatched cases, so $P_{matched} > P_{mismatched}$. We can express both numbers, again based on the cdf's of the distributions:

$P_{matched} = 0.5 * \left[1 + \mathrm{erf}\left(\frac{1}{\sigma_{soc} * \sqrt{2}}.\right)\right]$, where $\sigma_{soc}(i) = \frac{sd_{soc}}{\sqrt{p_i * M}}$; and

$P_{mismatched} = 0.5 * \left[1 + \mathrm{erf}\left(\frac{-1}{\sigma_{soc} * \sqrt{2}}.\right)\right]$, where $\sigma_{soc}(i) = \frac{sd_{soc}}{\sqrt{(1-p_i) * M}}$

The effectiveness of social learning depends on the number of times an individual of type *i* observes 'matched' and 'mismatched' traits (given by p_i and 1-p_i, respectively). We can calculate P_{soc} as the sum of the cdf's weighted by p_i:

$$P_{soc}(i) = p_i * P_{matched} + (1 - p_i) * p_{mismatched}$$

Figure SN2 summarises the distributions of P_{soc} for the same settings as those shown in Fig. 3b and c of the main text. We observe that increasing variation in payoffs leads to a decrease in average P_{soc} (Fig. SN2; white circles). In populations where polarisation is low, P_{soc} does not vary much across individuals. By contrast, when polarisation is high, there is a wide range of parameters in which some individuals strongly benefit from social learning (top of the panel; $P_{soc} > P_{ind}$), while for others social learning tends to lead to 'incorrect' decisions (bottom of panel; $P_{soc} < P_{ind}$). This means that when polarisation is strong, social learning is better for some individuals, while individual learning is better for others. These conditions create the scope for natural selection to favour developmental flexibility in social learning.

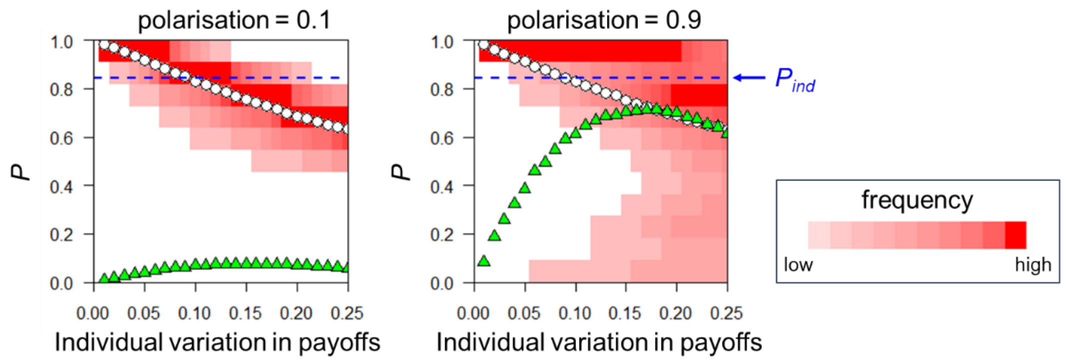

Figure SN2. Distributions of successful social learning as a function of individual variation in payoffs. The red shading indicates the frequencies of individuals for each level of P_{soc} (more opaque colours show higher frequencies). White circles and green triangles show average and variation in P_{soc},, respectively. The dashed blue line indicates P_{ind}.

Scenario 3

In Scenario 3 we extended Scenario 2 by adding assortment, which increases the probability that individuals learn from more similar others. This probability is based on the number of shared traits with positive payoffs. We can express the probability that an individual of type *i* learns from an individual of type *j* as $f_{ij} = (K_{ij} * n)^a$, where *a* is the assortment parameter (see Methods in main text for details).

When *a*=0, sampling is random, and with increasing *a*, individuals are increasingly more likely to learn from similar others. Fig. SN3 shows that assortment increases average P_{soc}, and that intermediate assortment is associated with the situation where there is substantial variation between individuals in whether social or individual learning is most effective (red shading respectively above and below the blue dashed line).

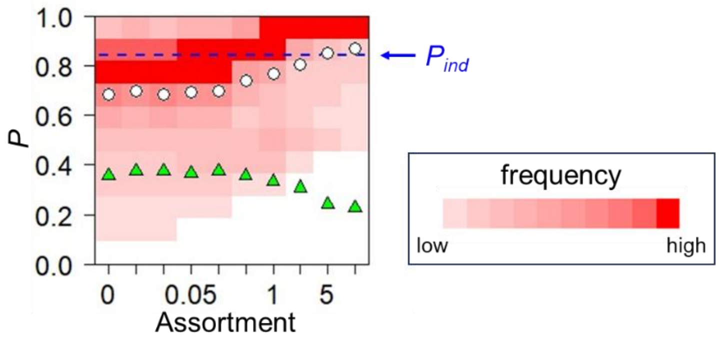

Fig. SN3. Distributions of successful social learning as a function of assortment. As in the main text, we assume that individual variation in payoffs is 0.2 and polarisation is 0.5.

Supplementary Figures

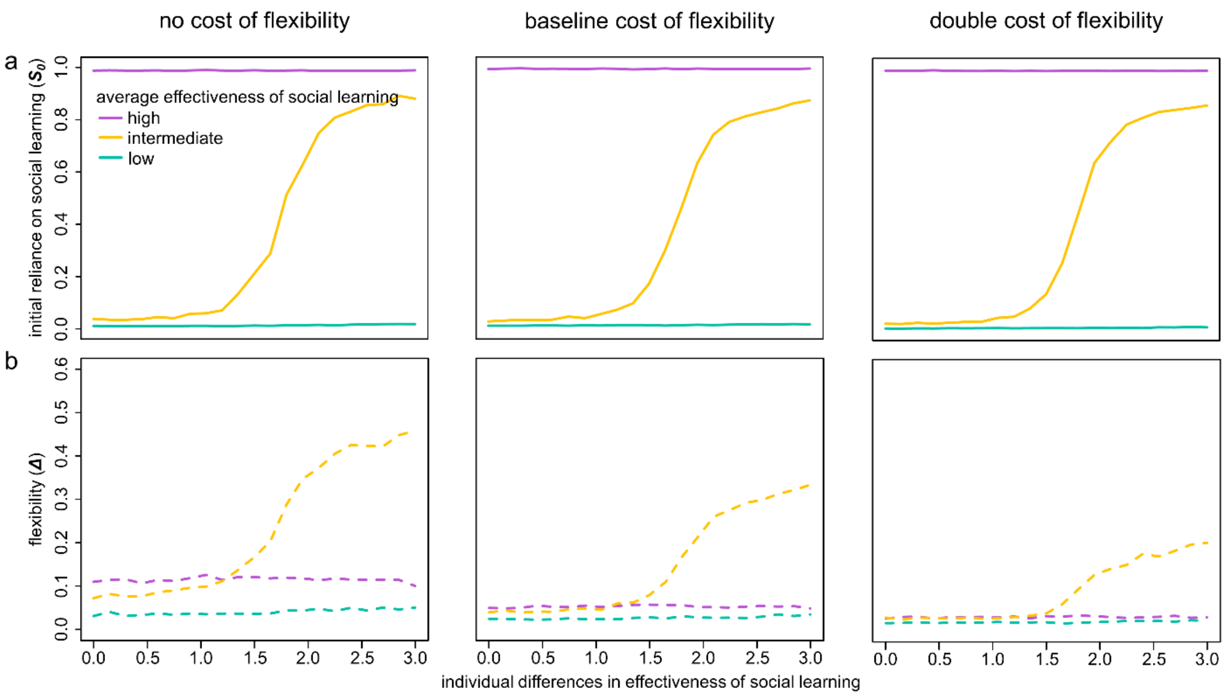

Fig S1. Flexibility in reliance on social learning evolves also when the cost of flexibility is absent or doubled. a, Evolved initial reliance on social learning (S_0) and **b**, flexibility in reliance on social learning (Δ), depending on the average (three lines) and variation (x-axis) in the effectiveness of social learning. Left panels show the situation where there is no fitness cost of flexibility, the middle panels show the situation where the cost is equal to the cost in the main text, and the panels on the right show the situation where the cost is twice as high as the cost in the main text.

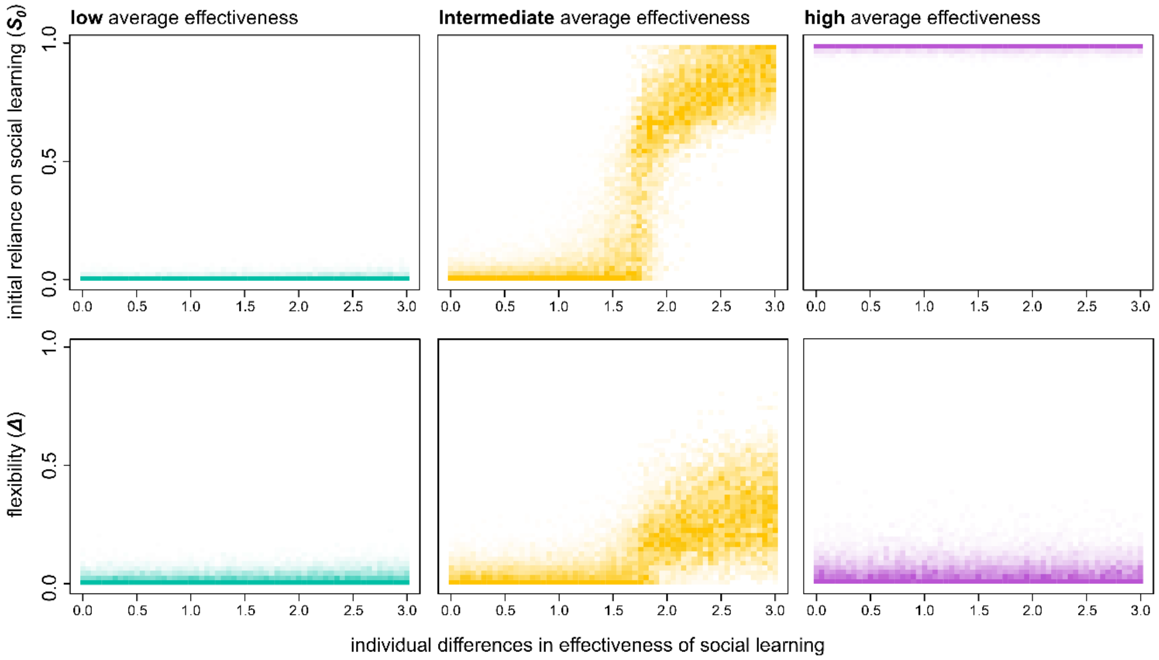

Fig S2. Full distributions of simulation outcomes across the entire range of individual differences in effectiveness of social learning. Evolutionary outcomes of 200 replicate simulations for each level of individual differences in effectiveness of social learning (x-axis). Shading indicates the number of simulations producing the corresponding outcomes (for initial reliance on social learning, top, and flexibility, bottom).

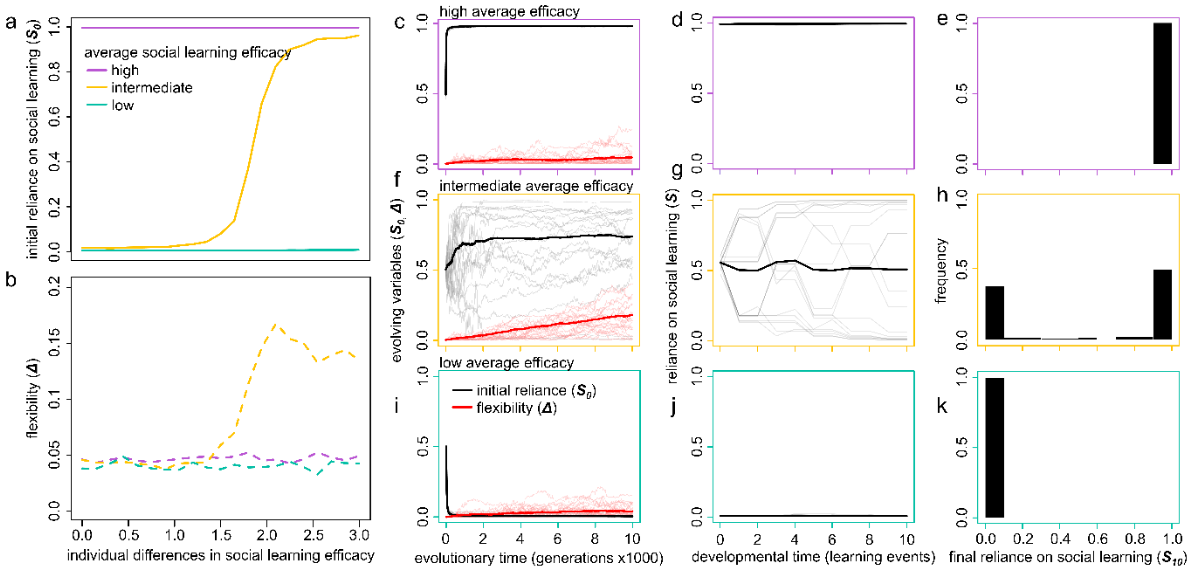

Fig S3. Flexibility and individual differences in reliance on social learning evolve when social learning pays for some but not for others, alternative implementation. The results shown are for the alternative implementation of the model described in the Supplementary Methods. Figure panels follow Fig. 2 of the main text. **a,** Evolved initial reliance on social learning (S_0) and **b**, flexibility in reliance on social learning (Δ), depending on the average (three lines) and variation (x-axis) in the effectiveness of social learning. Different mean degrees of effectiveness are indicated by different coloured lines. The degree of individual variation in effectiveness is on the horizontal axis. Lines indicate average outcomes over 200 replicate simulations for each parameter combination. **c**,**f**,**i**, Evolutionary trajectories over 10,000 generations of the population averages of initial reliance on social learning (S_0, black lines) and flexibility in reliance on social learning (Δ, red lines) for high (**c**), intermediate (**f**), and low (**i**) average social learning effectiveness. Graphs show evolutionary trajectories from 20 randomly chosen simulation replicates (thin lines) for each evolving variable, and their averages (thick lines). **d**,**g**,**j**, Individual developmental trajectories of reliance on social learning over 10 learning events, for high (**d**), intermediate (**g**), and low (**j**) social learning effectiveness. Graphs show trajectories of 20 randomly chosen individuals at the end of a single representative simulation, as well as the averages over these subsets of individuals (thick lines). **e**,**h**,**k**, Histograms of the reliance on social learning at the end of development (S_{10}). Bars show binned fractions of reliance on social learning across the entire population of the same simulation as the corresponding graphs d, g and j. For graphs c-k, the individual variation in social learning effectiveness is equal to 2.

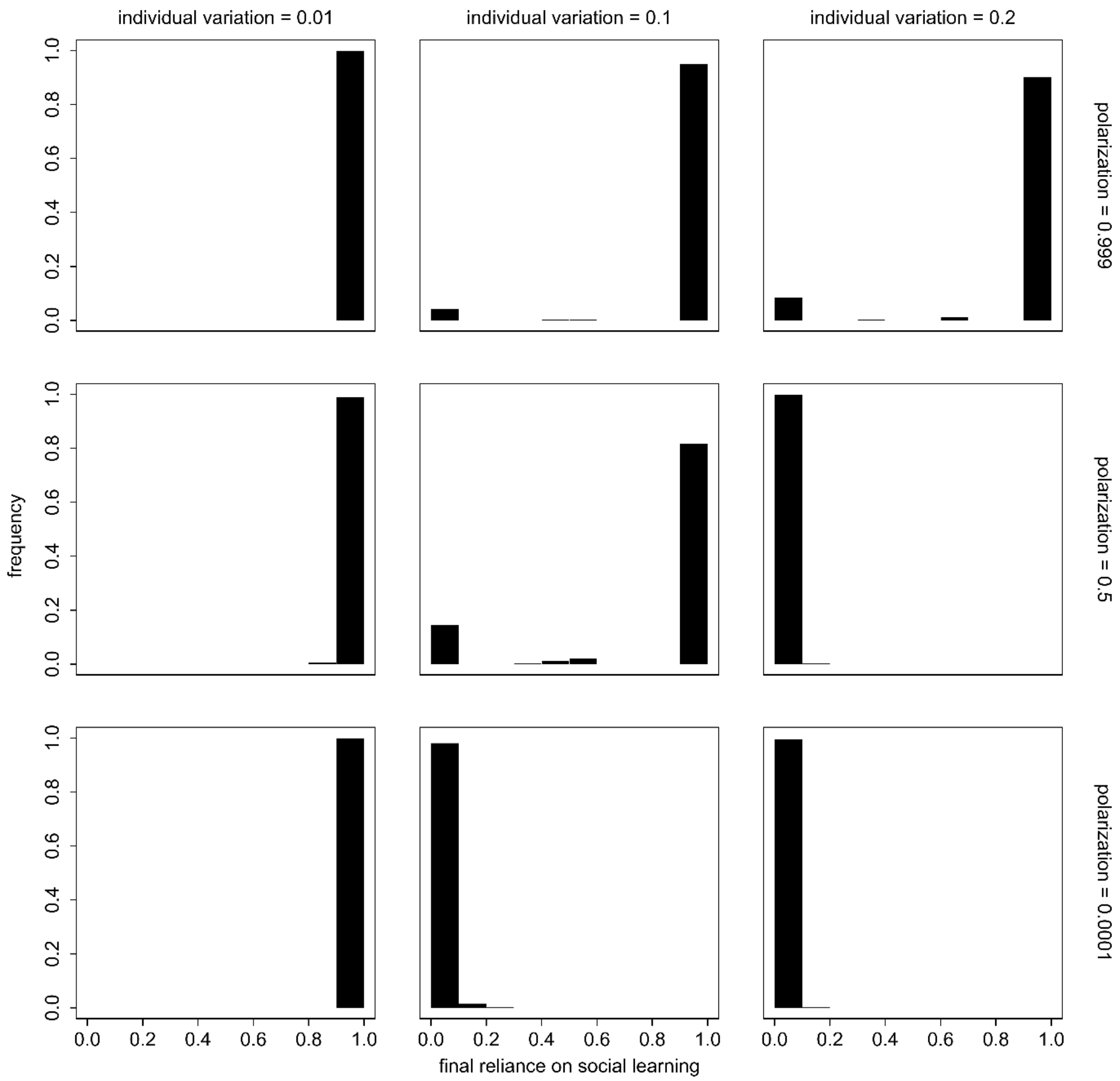

Fig S4. Histograms of the reliance on social learning at the end of development, depending on variation in payoffs that individuals receive from adopting cultural traits and the polarisation of these differences. Bars show binned fractions of reliance on social learning across the entire population of a single representative simulation.

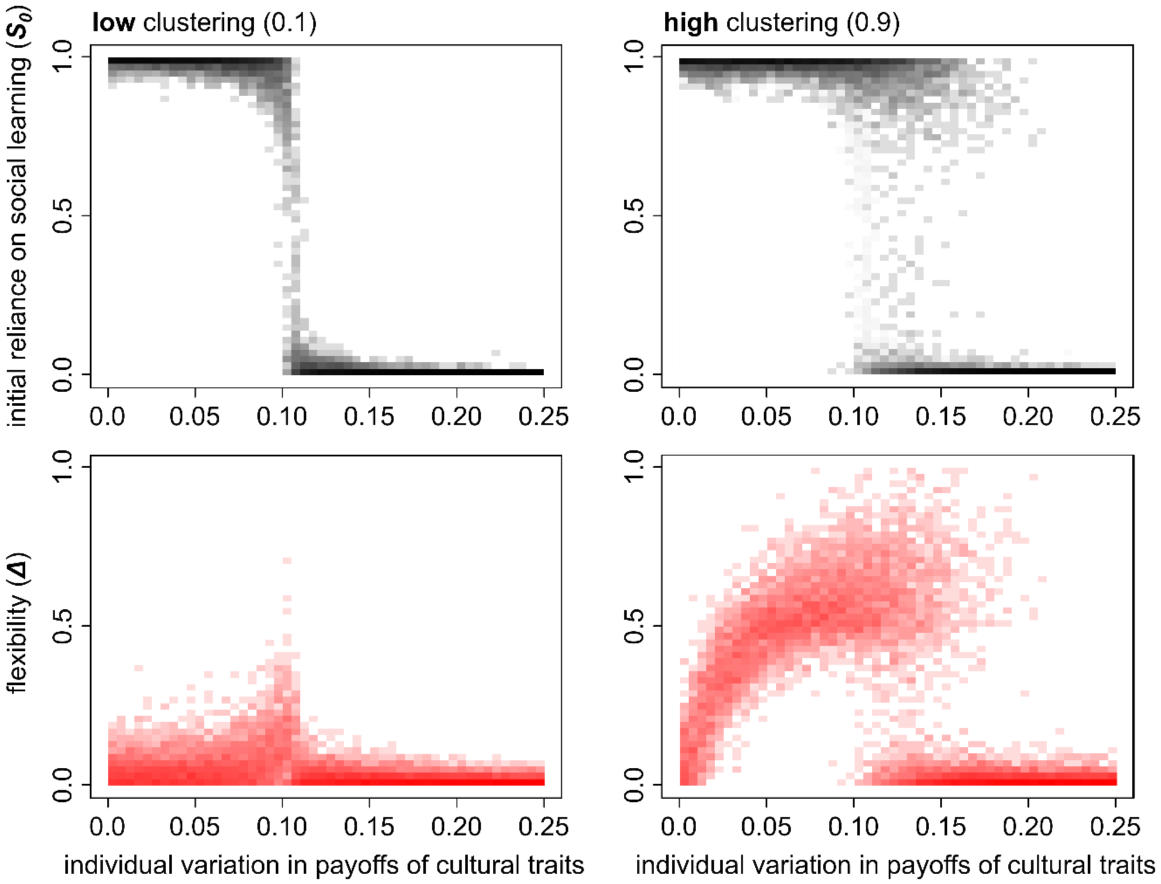

Fig S5. The evolution of flexibility in reliance on social learning when individuals obtain different payoffs from adopting cultural traits. Evolutionary outcomes of 200 replicate simulations for each level of individual variation in payoffs of cultural traits (x-axis). Shading indicates the number of simulations producing the corresponding outcomes (for initial reliance on social learning, top, and flexibility, bottom). Graphs show results for initial reliance on social learning and flexibility when clustering is low (0.1; leftmost graphs) and high (rightmost graphs).

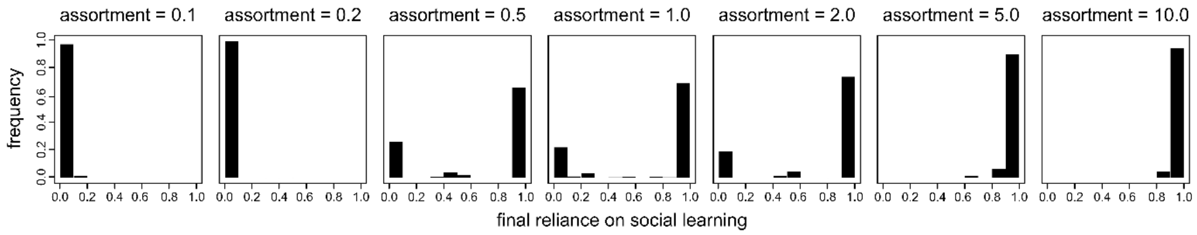

Fig S6. Histograms of the reliance on social learning at the end of development, depending on assortment. Bars show binned fractions of reliance on social learning across the entire population of a single representative simulation. For these simulations, the individual variation in social learning is equal to 0.2 and the polarisation is 0.5 (as in Figure 4).

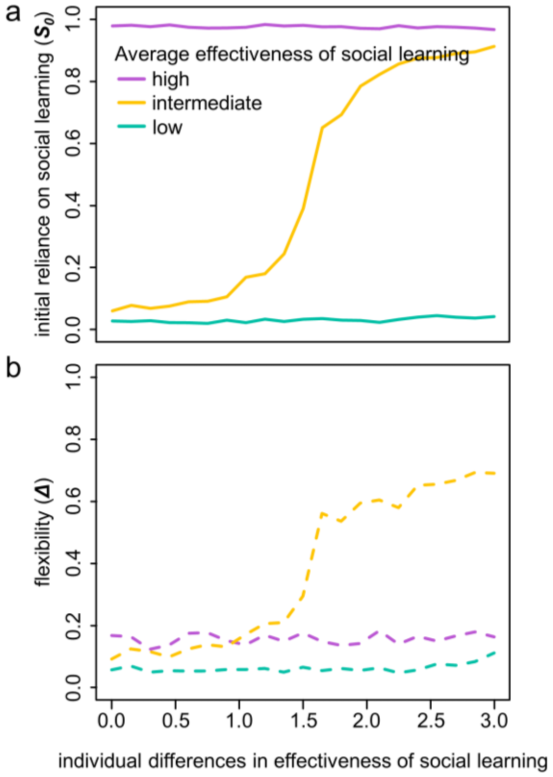

Fig S7. Flexibility in reliance on social learning evolves also when mutations are independent of the parent genotype. a, Evolved initial reliance on social learning (S_0) and **b**, flexibility in reliance on social learning (Δ), depending on the average (three lines) and variation (x-axis) in the effectiveness of social learning. These simulations had a different implementation of mutation compared to the ones shown in Figure 1 of the main text: when a mutation occurred, the new value of the gene was set equal to a randomly drawn number from a uniform distribution between 0 and 1 (for both S_0 and Δ), and so was no longer dependent on the parental value of the gene.

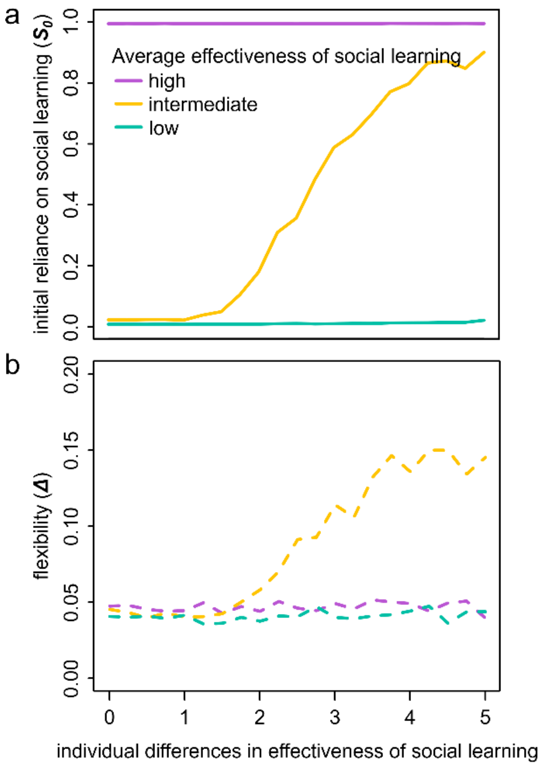

Fig S8. Flexibility in reliance on social learning evolves also when payoffs continuously vary around -1 and 1. a, Evolved initial reliance on social learning (S_0) and **b**, flexibility in reliance on social learning (Δ), depending on the average (three lines) and variation (x-axis) in the effectiveness of social learning. For these simulations, rather than setting all payoffs of odd cultural traits equal to 1 and all payoffs of even cultural traits equal to -1, we added some noise drawn from a normal distribution with the mean centred on respectively 1 and -1, and standard deviation equal to 0.5. The payoffs were drawn once at the start of the simulation and were equal for all individuals in all generations. Individuals update their reliance on social learning according to the model described in the Supplementary Methods (so they are sensitive to the magnitude of the payoffs in updating, which the model described in the main text does not allow for).

Supplementary References

1. Wagner, A. R. & Rescorla, R. A. Inhibition in Pavlovian conditioning: Application of a theory. *Inhib. Learn.* 301–336 (1972).
