# Supplementary material for: Unpredictable benefits of social information can lead to the evolution of individual differences in social learning
Source: Nat Commun. 2024 Jun 15;15:5138. doi: 10.1038/s41467-024-49530-4 (PMC11180142; doi:10.1038/s41467-024-49530-4)
Supplement: Supplementary file 3 — Reporting Summary [file 41467_2024_49530_MOESM3_ESM.pdf]

nature portfolio

Corresponding author(s): Pieter van den Berg

Last updated by author(s): 26-04-2024

Reporting Summary

Nature Portfolio wishes to improve the reproducibility of the work that we publish. This form provides structure for consistency and transparency in reporting. For further information on Nature Portfolio policies, see our Editorial Policies and the Editorial Policy Checklist.

Statistics

For all statistical analyses, confirm that the following items are present in the figure legend, table legend, main text, or Methods section.

n/a	Confirmed	
☐	☒	The exact sample size (*n*) for each experimental group/condition, given as a discrete number and unit of measurement
☐	☒	A statement on whether measurements were taken from distinct samples or whether the same sample was measured repeatedly
☒	☐	The statistical test(s) used AND whether they are one- or two-sided<br>*Only common tests should be described solely by name; describe more complex techniques in the Methods section.*
☒	☐	A description of all covariates tested
☒	☐	A description of any assumptions or corrections, such as tests of normality and adjustment for multiple comparisons
☐	☒	A full description of the statistical parameters including central tendency (e.g. means) or other basic estimates (e.g. regression coefficient) AND variation (e.g. standard deviation) or associated estimates of uncertainty (e.g. confidence intervals)
☒	☐	For null hypothesis testing, the test statistic (e.g. *F*, *t*, *r*) with confidence intervals, effect sizes, degrees of freedom and *P* value noted<br>*Give P values as exact values whenever suitable.*
☒	☐	For Bayesian analysis, information on the choice of priors and Markov chain Monte Carlo settings
☒	☐	For hierarchical and complex designs, identification of the appropriate level for tests and full reporting of outcomes
☒	☐	Estimates of effect sizes (e.g. Cohen's *d*, Pearson's *r*), indicating how they were calculated

Our web collection on statistics for biologists contains articles on many of the points above.

Software and code

Policy information about availability of computer code

Data collection	All simulation and analysis software is available in the publicly accessible repository associated with our paper: https://osf.io/7ta9m
Data analysis	All simulation and analysis software is available in the publicly accessible repository associated with our paper: https://osf.io/7ta9m

For manuscripts utilizing custom algorithms or software that are central to the research but not yet described in published literature, software must be made available to editors and reviewers. We strongly encourage code deposition in a community repository (e.g. GitHub). See the Nature Portfolio guidelines for submitting code & software for further information.

Data

Policy information about availability of data

All manuscripts must include a data availability statement. This statement should provide the following information, where applicable:

- Accession codes, unique identifiers, or web links for publicly available datasets
- A description of any restrictions on data availability
- For clinical datasets or third party data, please ensure that the statement adheres to our policy

The simulation data generated in this study have been deposited in a repository of the Open Science Framework: https://osf.io/7ta9m/

Research involving human participants, their data, or biological material

Policy information about studies with human participants or human data. See also policy information about sex, gender (identity/presentation), and sexual orientation and race, ethnicity and racism.

Reporting on sex and gender	N/A
Reporting on race, ethnicity, or other socially relevant groupings	N/A
Population characteristics	N/A
Recruitment	N/A
Ethics oversight	N/A

Note that full information on the approval of the study protocol must also be provided in the manuscript.

Field-specific reporting

Please select the one below that is the best fit for your research. If you are not sure, read the appropriate sections before making your selection.

☐ Life sciences ☐ Behavioural & social sciences ☒ Ecological, evolutionary & environmental sciences

For a reference copy of the document with all sections, see nature.com/documents/nr-reporting-summary-flat.pdf

Ecological, evolutionary & environmental sciences study design

All studies must disclose on these points even when the disclosure is negative.

Study description	Individual-based simulations to study the evolution of 2 learning parameters: (i) initial reliance on social learning and (ii) flexibility in reliance on social learning. In three sets of simulations we vary (a effecctiveness of social learning; (b) payoff profiles; and (c) degrees of assortment. Each simulation run represents an independent replication of the evolutionary process under a set of conditions. For each parameter combination that we consider, we report summary statistics of 200 independent simulations runs.
Research sample	Individual-based simulations conducted in C++.
Sampling strategy	For each parameter combination that we consider, we report summary statistics of 200 independent simulations runs. For the illustration purposes in Fig. 2c,f,i, we show 20 randomly chosen simulation runs, and in Fig 2d,g,j, we shown 20 randomly chosen individuals.
Data collection	Summary statistics taken from the simulations.
Timing and spatial scale	Simulations were run in July 2023; with some additional simulations in December 2023 to address reviewer comments.
Data exclusions	No data was excluded from analysis.
Reproducibility	The data and simulation code are publicly available via https://osf.io/7ta9m
Randomization	Does not apply in this simulation study.
Blinding	Does not apply in this simulation study.

Did the study involve field work? ☐ Yes ☒ No

Reporting for specific materials, systems and methods

We require information from authors about some types of materials, experimental systems and methods used in many studies. Here, indicate whether each material, system or method listed is relevant to your study. If you are not sure if a list item applies to your research, read the appropriate section before selecting a response.

Materials & experimental systems

n/a	Involved in the study
☒	☐ Antibodies
☒	☐ Eukaryotic cell lines
☒	☐ Palaeontology and archaeology
☒	☐ Animals and other organisms
☒	☐ Clinical data
☒	☐ Dual use research of concern
☒	☐ Plants

Methods

n/a	Involved in the study
☒	☐ ChIP-seq
☒	☐ Flow cytometry
☒	☐ MRI-based neuroimaging

Plants

Seed stocks	N/A
Novel plant genotypes	N/A
Authentication	N/A
